# Conditional Deletion of Cytoplasmic Dynein Heavy Chain in Postnatal Photoreceptors

**DOI:** 10.1167/iovs.62.14.23

**Published:** 2021-11-22

**Authors:** Tiffanie M. Dahl, Michelle Reed, Cecilia D. Gerstner, Wolfgang Baehr

**Affiliations:** 1 Department of Ophthalmology, University of Utah Health Science Center, Salt Lake City, Utah, United States; 2 Department of Neurobiology & Anatomy, University of Utah, Salt Lake City, Utah, United States; 3 Department of Biology, University of Utah, Salt Lake City, Utah, United States

**Keywords:** dynein-1, dynein heavy chain, conditional knockout

## Abstract

**Purpose:**

Cytoplasmic dynein-1 (henceforth dynein) moves cargo in conjunction with dynactin toward the minus end of microtubules. The dynein heavy chain, DYNC1H1, comprises the backbone of dynein, a retrograde motor. Deletion of *Dync1h1* abrogates dynein function. The purpose of this communication is to demonstrate effects of photoreceptor dynein inactivation during late postnatal development and in adult retina.

**Methods:**

We mated *Dync1h1*^F/F^ mice with iCre75 and Prom1-CreER^T2^ mice to generate conditional rod and tamoxifen-induced knockout in rods and cones, respectively. We documented retina degeneration with confocal microscopy at postnatal day (P) 10 to P30 for the iCre75 line and 1 to 4 weeks post tamoxifen induction (wPTI) for the Prom1-CreER^T2^ line. We performed scotopic and photopic electroretinography (ERG) at P16 to P30 in the iCre75 line and at 1-week increments in the Prom1-CreER^T2^ line. Results were evaluated statistically using Student's *t*-test, two-factor ANOVA, and Welch's ANOVA.

**Results:**

Cre-induced homologous recombination of Dync1h1^F/F^ mice truncated DYNC1H1 after exon 23. ^rod^*Dync1h1*^−/−^ photoreceptors degenerated after P14, reducing outer nuclear layer (ONL) thickness and combined inner segment/outer segment (IS/OS) length significantly by P18. Scotopic ERG a-wave amplitudes decreased by P16 and were extinguished at P30. Cones were stable under rod-knockout conditions until P21 but inactive at P30. In ^tam^*Dync1h1*^−/−^ photoreceptors, the IS/OS began shortening by 3wPTI and were nearly eliminated by 4wPTI. The ONL shrank significantly over this interval, indicating rapid photoreceptor degeneration following the loss of dynein.

**Conclusions:**

Our results demonstrate dynein is essential for the secretory pathway, formation of outer segments, and photoreceptor maintenance.

Transport along microtubules is accomplished by cytoplasmic dynein,^1^ which moves cargo toward the minus end of microtubules at the basal body.^2^^–^^4^ In photoreceptors, the minus end is located at the basal body, serving as a microtubule organizing center (MTOC) (for review, see Baehr et al.^5^). During early photoreceptor development, dynein cargo may include nuclei,^6^ mitochondria,^4^^,^^7^ membrane vesicles,^8^^,^^9^ and other organelles (reviewed in Reck-Peterson et al.^10^). After ciliogenesis, dynein plays a key role in the secretory pathway transporting membrane vesicles and membrane proteins from the endoplasmic reticulum to the periciliary ridge, where cargo is unloaded.^11^

Dynein is composed of a pair of heavy chains (DYNC1H1) (Fig. 1A) and a set of noncatalytic accessory components termed intermediate, light intermediate, and light chains.^12^^–^^14^ DYNC1H1 serves as a scaffold organizing the distribution of dynein subunits and the three-dimensional structure of the dynein megacomplex (Fig. 1B).^15^^,^^16^ The N-terminal DYNC1H1 tail domain contains a dimerization domain^17^ and binding sites for intermediate chains (DIC1 or DIC2) and light intermediate chains (DLIC1 or DLIC2). The C-terminal half contains the motor domain and microtubule interaction site. The dynein motor domain is built around a ring of six AAA+ (ATPases Associated with various Activities) of the heavy chain. The microtubule-binding domain sits at the tip of a coiled-coiled stalk emerging from AAA4. Dynactin and a cargo adaptor are essential for cytoplasmic dynein to move membrane vesicles along microtubules.^18^

**Figure 1.**
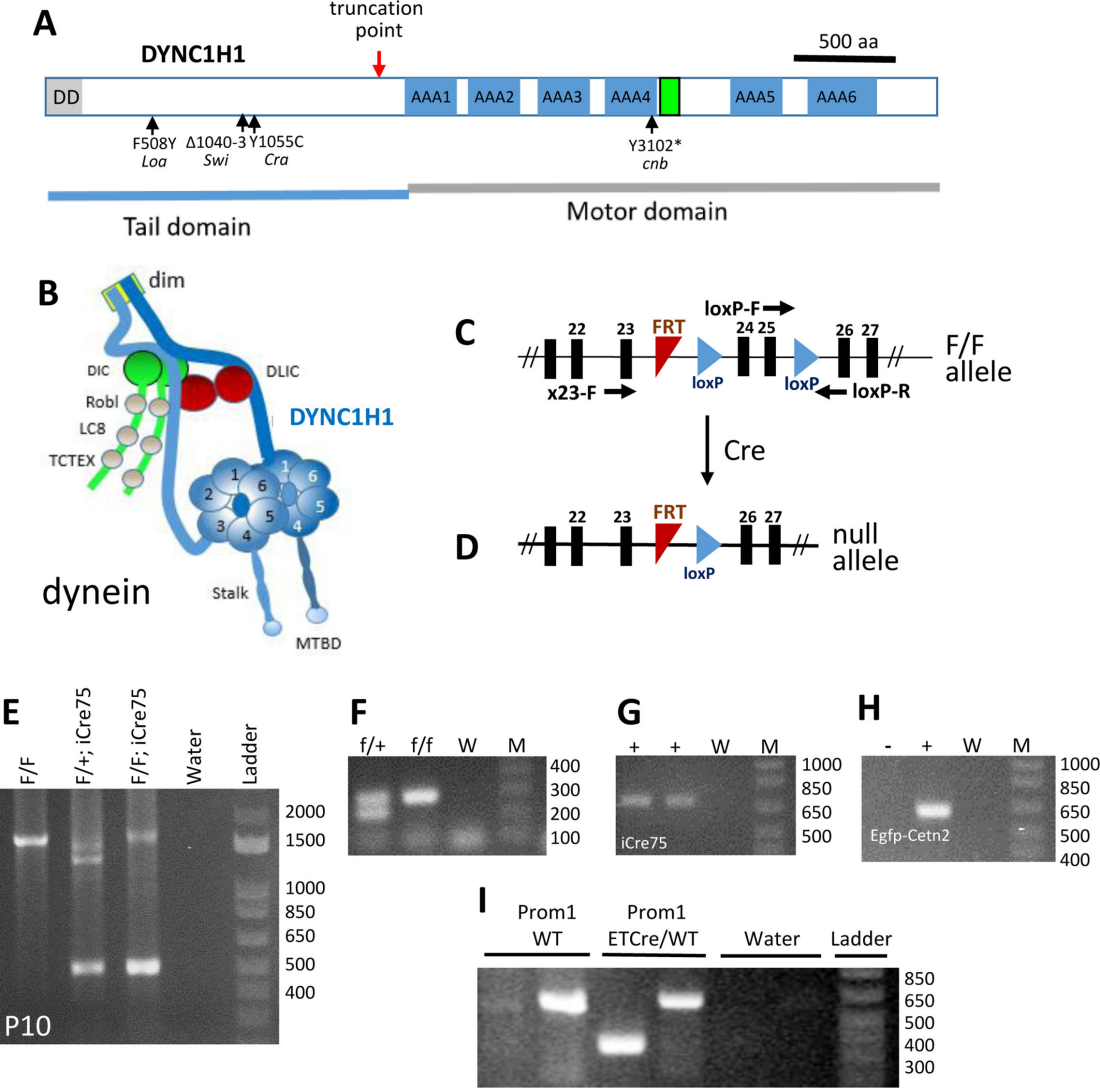
Photoreceptor-specific knockout of *Dync1h1*. (**A**) Schematic of mouse cytoplasmic dynein heavy chain (DYNC1H1, gene symbol *Dync1h1*). Locations of the truncation point (*red arrow*), six AAA motor domains (*blue*), and microtubule-binding domain (*green*) are indicated. Locations of previously identified mutations in mice and zebrafish are shown below the protein. *Dync1h1* knockout results in truncation of DYNC1H1 in its tail domain and eliminates the motor domain. (**B**) Cytoplasmic dynein drawn schematically with heavy chain (*blue*). Adapted from Hoang et al.^22^ (**C**) loxP sites of the floxed *Dync1h1* allele are located in introns 23 and 25. (**D**) Following Cre-induced recombination, the null allele loses exons 24 and 25. (**E**) PCR amplification using P10 retinal DNA as template and primers X23F and loxP-R. The amplicon at 500 bp shows Cre-induced recombination has occurred in the *Dync1h1*^F/F^;iCre75 retina, while amplicon at 1600 bp represents *Dync1h1*^F/F^ present in the inner retina. (**F**) Genotyping of *Dync1h1*^F/F^ and *Dync1h1*^F/+^ alleles with primers loxP-F and loxP-R in intron 25. M, size markers; W, water control. (**G**) Genotyping of iCre75. +, tail DNA containing the iCre75 transgene. (**H**) Genotyping of Egfp-Cetn2. +, tail DNA containing the Egfp-Cetn2 transgene. (**I**) Genotyping of Prom1-ETCre mice.

Numerous mutations in the human *DYNC1H1* gene associate with spinal muscular atrophy and Charcot–Marie–Tooth disease.^19^^–^^23^ Germline deletion of mouse *Dync1h1* (truncation after exon 1) is lethal as embryos do not survive beyond embryonic day 8.5 (E8.5).^24^ Four mouse mutants—legs at odd angles (*Loa*),^25^^,^^26^ Cramping 1 (*Cra1*),^27^^,^^28^ Sprawling (*Swl*),^29^^,^^30^ and a model of Charcot–Marie–Tooth disease^31^^,^^32^—display autosomal dominant behavioral phenotypes with no reported retina phenotype. A DYNC1H1 nonsense mutation (Y3102X) underlies the *cannonball* (*cnb*) phenotype in zebrafish.^4^ Green fluorescent protein (GFP)–tagged rhodopsin has been mislocalized in both the *cannonball* mutant and *dync1h1* morphant cells.^4^

Dynein is present prominently in photoreceptor inner segments (ISs), the outer plexiform layer (OPL), and the inner plexiform layer, as well as, to a lesser extent, within the outer nuclear layer (ONL), inner nuclear layer (INL), and ganglion cell layer. Dynein is responsible for retina lamination, nuclear positioning, and vesicular trafficking of photoreceptor membrane proteins.^13^^,^^33^ We previously reported that conditional knockout of Dync1h1 in mouse retina (*Dync1h1*^F/F^;Six3cre or ^ret^*Dync1h1*^−/−^) resulted in rapid retinal degeneration within 2 postnatal weeks.^13^ DYNC1H1 was undetectable in the ^ret^*Dync1h1*^−/−^ IS area as early as postnatal day (P) 6, and photoreceptors did not elaborate IS, outer segment (OS), or functional synaptic terminals. In the P8 ^ret^*Dync1h1^−^*^/−^ central retina, outer and inner nuclear layers were severely disorganized and lacked a recognizable OPL.^13^

In this report, we depleted DYNC1H1 in rod photoreceptors during postnatal development and in both rods and cones of adult mice by tamoxifen induction. These knockouts permit analysis of the consequences of DYNC1H1 deletion during late postnatal development in rods and in photoreceptors of adult mice, respectively.

## Results

### Generation of DYNC1H1 Conditional Knockout Mice

The dynein heavy chain DYNC1H1 (Fig. 1A) forms the backbone of cytoplasmic dynein, a mega complex of 1.5 mDa, composed of a pair of force-generating heavy chains and a set of accessory components termed intermediate, light intermediate, and light chains (Fig. 1B). The heavy chain serves as a scaffold organizing the distribution of dynein subunits. We previously generated a floxed *Dync1h1* allele (*Dync1h1*^F/F^), in which loxP sites are placed in introns 23 and 25 (Fig. 1C).^13^ Deletion of exons 24 and 25 truncates DYNC1H1 (red arrow, Fig. 1A, D) removing the C-terminal motor domain and the microtubule binding site. We mated *Dync1h1*^F/F^ mice with iCre75 transgenic mice to generate rod knockout mice (*Dync1h1*^F/F^;iCre75, abbreviated ^rod^*Dync1h1*^−/−^) and with CreER^T2^ mice (*Dync1h1*^F/F^;Prom1-CreER^T2^) to generate rod/cone knockouts following tamoxifen induction (^tam^*Dync1h1*^−/−^). In iCre75 mice, Cre is expressed under the control of a rhodopsin promoter, which drives Cre in the second postnatal week.^34^ In Prom1-CreER^T2^ mice, CreERT2 expression occurs under the control of the Prom1 promoter driving expression in rods and cones.^35^ Deletion of the *Dync1h1* gene in ^rod^*Dync1h1*^−/−^ mice was assessed by PCR with P10 retina DNA as template and primers X23-F and loxP-R to generate a 500-bp amplicon lacking exons 24/25 (Fig. 1E). Since dynein is expressed in all retina cells, a band at 1.6 kb corresponding to the *Dync1h1*^F^ allele is also observed. We did not perform an immunoblot as a blot using total retina tissue would be inconclusive due to the continued presence of DYNC1H1 in all nonrod retina cells. Genotyping with tail DNA confirmed the presence of loxP (indicating a *Dync1h1*^F^ allele) (Fig. 1F), iCre75 (Fig. 1G), EGFP-CETN2 (Fig. 1H), and Prom1-CreER^T2^ (Fig. 1I) in respective mice (see Methods for details).

### Electroretinography of Rod Knockouts

**Figure 2.**
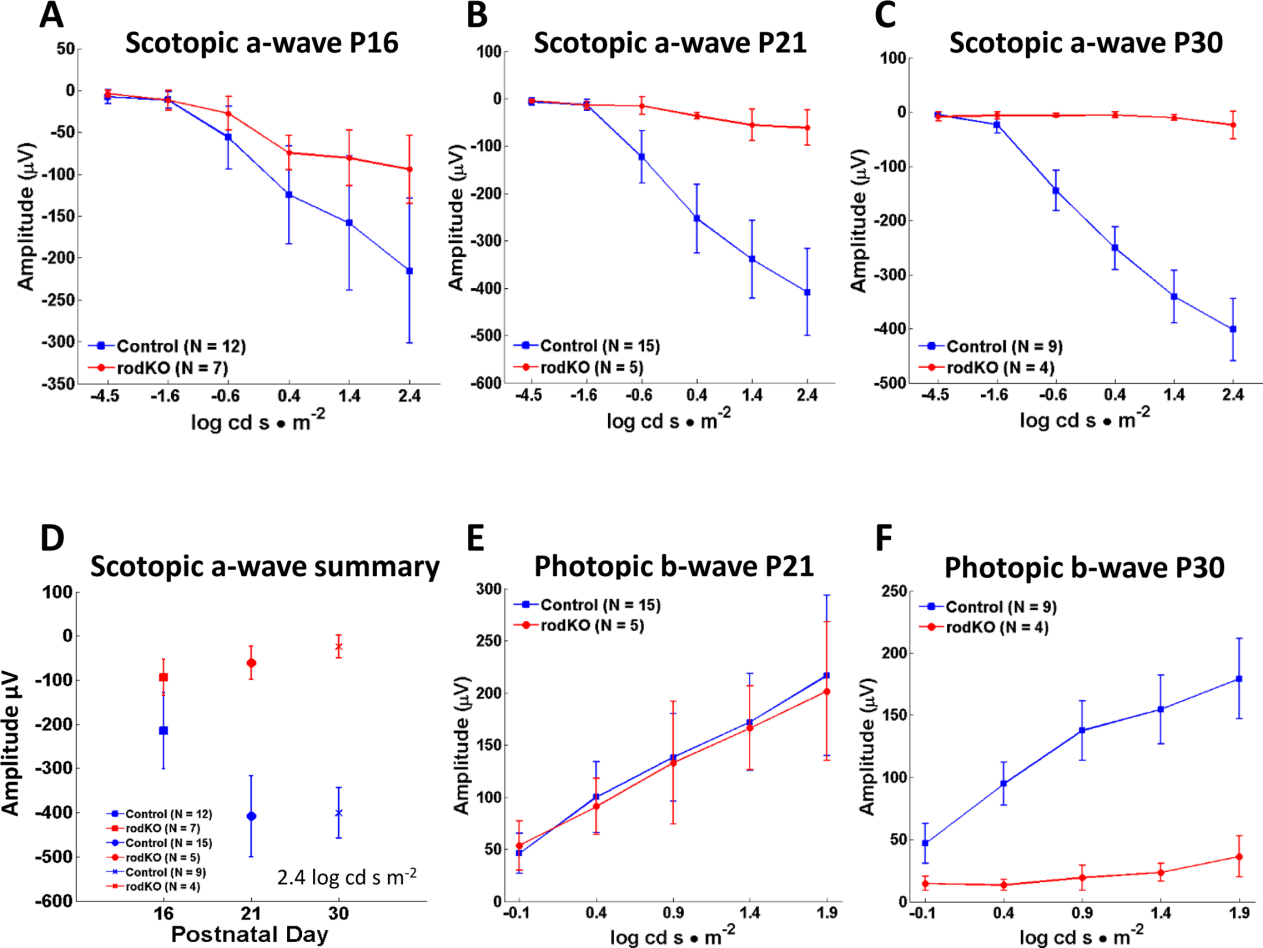
Scotopic and photopic electroretinography. (**A–C**) Average scotopic a-wave amplitudes as a function of light intensity (−4.5 to 2.4 log cd s·m^−2^) at P16 (A), P21 (B), and P30 (C). The scotopic ^rod^*Dync1h1^−^^/^^−^* a-wave amplitude decreases by P21 and is nearly extinguished at P30. (**D**) Summary of peak amplitudes at 2.4 log cd s·m^−2^; average peak scotopic a-wave amplitude and standard deviations are indicated. (**E**, **F**) Average photopic b-wave amplitudes as a function of light intensity (−0.1 to 1.9 log cd s·m^−2^) at P21 (E) and P30 (F). Decline of the P30 photopic b-wave is probably caused by bystander cone degeneration following loss of rods in the rod knockout.

Scotopic electroretinography as a function of light intensity showed diminished a-wave amplitudes at P16 (Figs. 2A, D), suggesting rod degeneration had begun. A-wave amplitudes were further attenuated at P21 (Fig. 2B) and essentially extinguished at P30 (Figs. 2C, D), indicating that rod photoreceptor degeneration progressed rapidly and was completed 2 weeks after onset. We assessed cone function in the DYNC1H1 rod knockout by assessing the photopic b-wave. Photopic b-wave amplitudes of control and rod knockout mice were similar at P21 (Fig. 2E) but were extinguished at P30 (Fig. 2F). Assuming that cone bipolar cells are unaffected in the dynein rod knockout, the loss of photopic b-wave results from loss of cone function. This is consistent with previous findings in which deletion of rod-specific genes resulted in secondary loss of cones due to diminished rod-derived cone viability factor.^36^ Examples of cone loss following loss of rods have been documented in animal models of dominant or recessive retinitis pigmentosa (RP), the *rd1* mouse,^37^ and bystander cone degeneration of human RP.^38^

### Rapid Rod Degeneration in the Absence of DYNC1H1

**Figure 3.**
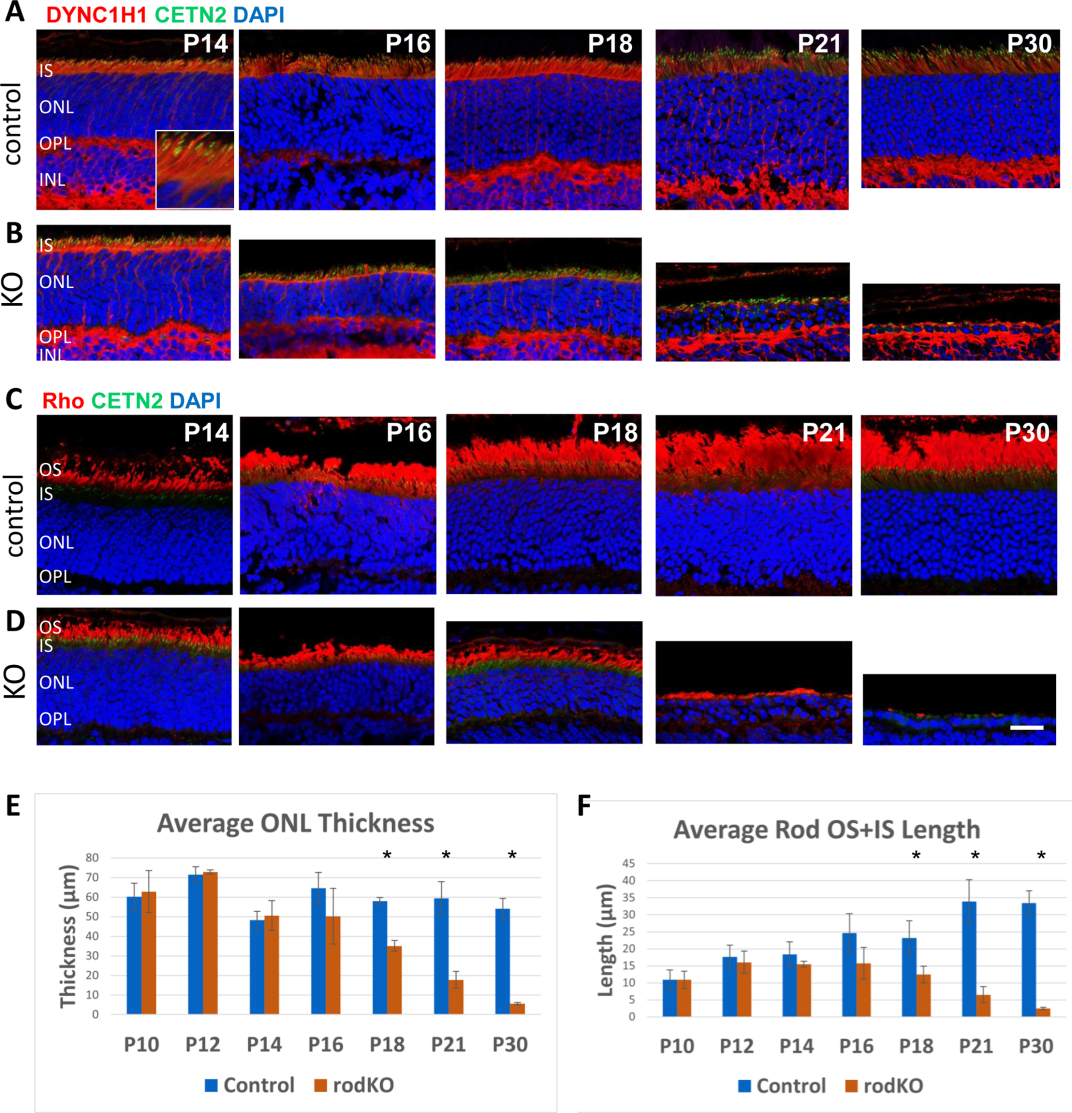
Immunohistochemistry with DYNC1H1 and rhodopsin (Rho) antibodies. (**A–D**) Representative control (rows A, C) and ^rod^*Dync1h1*^−/−^ retinas (rows B, D) labeled with antibodies directed against DYNC1H1 (rows A, B) and rhodopsin (rows C, D) at P14, P16, P18, P21, and P30. DYNC1H1 is located in the photoreceptor IS and OPL (photoreceptor terminals and bipolar cell dendrites). The ^rod^*Dync1h1^−^^/^^−^* ONL starts to shrink at P16, and only cone nuclei remain at P30. Rhodopsin-containing rod OSs are severely shortened by P21 and absent by P30. Note, almost no rhodopsin accumulates in the ONL. *Scale bar*: 20 µm. (**E**) Comparison of the average ONL thickness of control and ^rod^*Dync1h1*^−/−^ central retinas illustrates loss of rod photoreceptors between P16 and P30. *Error bars* show the standard deviation. *P < 0.05 (Student's *t*-test). (**F**) Comparison of the average combined length of rod OS + IS for P10 to P30 control and ^rod^*Dync1h1*^−/−^ retinas shows loss of OS and IS in the absence of DYNC1H1. *Error bars* show the standard deviation. *P < 0.05 (Student's *t*-test). For E and F, the numbers of mice for control and rodKO, respectively, were P10, *n* = 5, 4; P12, *n* = 8, 3; P14, *n* = 5, 3; P16, *n* = 8, 3; P18, *n* = 3, 3; P21, *n* = 7, 3; P30, *n* = 10, 3.

While DYNC1H1 was present in all retina cells, the highest concentrations were found in the photoreceptor IS, OPL, and, to a lesser extent, the ONL (Fig. 3, row A). Our iCre75 mice were maintained on the Egfp-Cetn2^+^ background, in which centrin 2 (CETN2) inhabited the connecting cilium tethering the IS to OS (Fig. 3A, see P14 inset) and aided to identify photoreceptor compartments. Although the *Dync1h1* gene was knocked out in rods by P10 (Fig. 1E), DYNC1H1 protein persisted in the rod ONL/IS (Fig. 3, row B), and thus ^rod^*Dync1h1*^−/−^ morphology was indistinguishable from controls at P14. DYNC1H1 levels were reduced in the IS beginning at P16, but some DYNC1H1 was still detectable at P30, presumably in cone inner segments (Fig. 3, rows B). DYNC1H1 in the OPL persisted as distal processes of rod bipolar cells were unaffected. ^rod^*Dync1h1*^−/−^ IS/OS regions and ONL began to shrink after P16 (Figs. 3E, F). The mutant ONL revealed three to four layers of nuclei at P21, and only a single nuclear layer (presumably cones) was evident at P30. Unexpectedly, we observed little rhodopsin accumulation or mislocalization in the ^rod^*Dync1h1*^−/−^ ONL (Fig. 3D).

### OS Protein PDE6 and Synaptic Protein CtBP2/RIBEYE in Rod Knockouts

**Figure 4.**
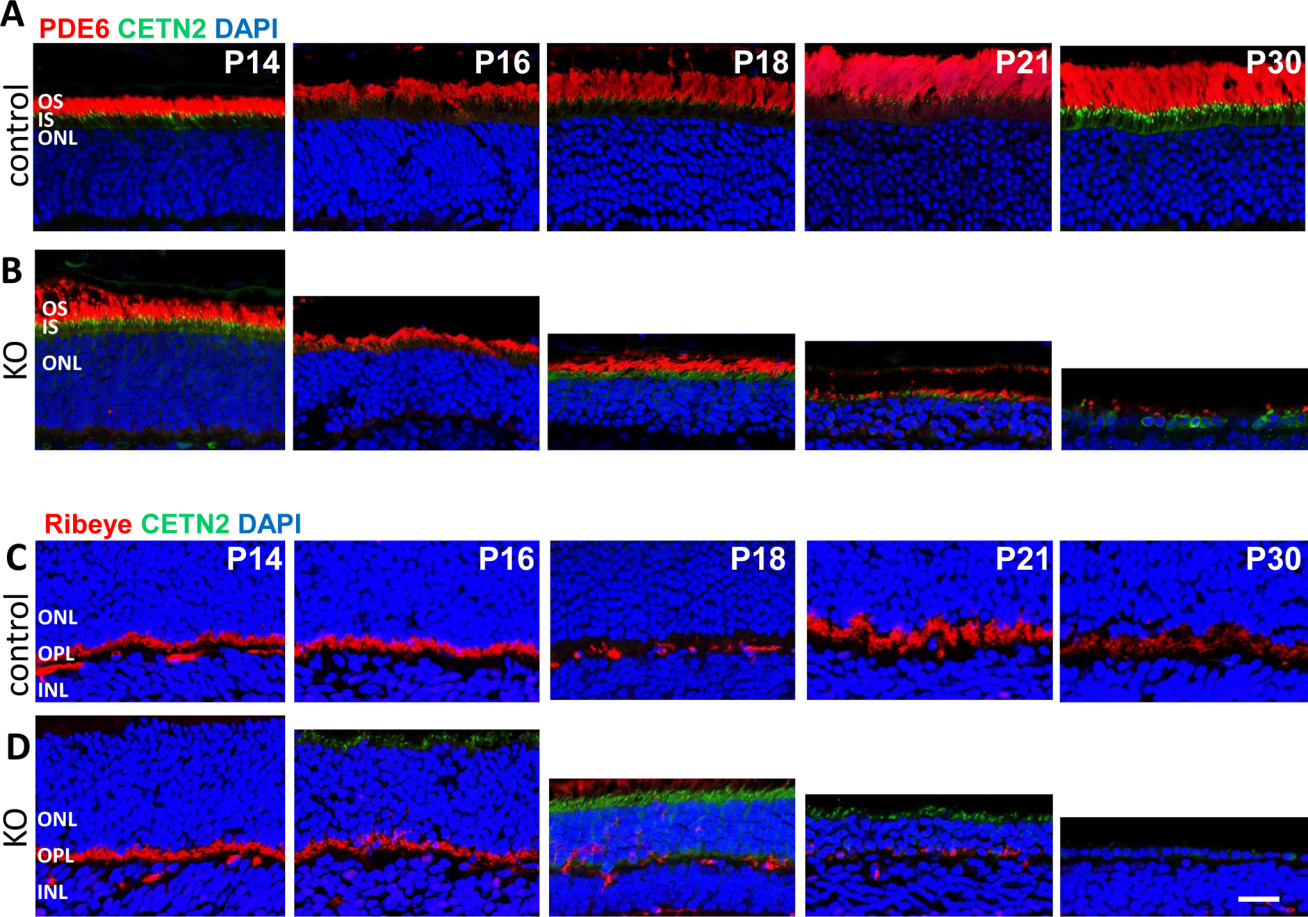
Immunohistochemistry with PDE6 and RIBEYE antibodies. (**A–D**) Representative control (rows A, C) and ^rod^*Dync1h1*^−/−^ retinas (rows B, D) labeled with antibodies directed against PDE6 (MOE; rows A, B) and RIBEYE (rows C, D). Rod and cone OS are shortened by P16 and lost by P30. RIBEYE expression in the ribbon synapses is lost between P16 and P30 with diminished expression evident by P21.

We determined the fates of rod PDE6 and RIBEYE, both of which are not directly dependent on dynein for transport to their destinations (OS and synapse, respectively). Rod PDE6 is a heterotetrameric protein consisting of isoprenylated PDE6α and PDE6β catalytic subunits and two PDE6γ inhibitory subunits (reviewed in Cote^39^). Subunit delivery to the OS is dependent on diffusion by interaction with PDE6D, a solubilization factor. In the P16 ^rod^*Dync1h1*^−/−^ retina, PDE6 content was reduced significantly based on OS shortening (Fig. 4, row B). As observed with rhodopsin, PDE6 never accumulated in the IS during OS shrinking at P16 and P18.

RIBEYE, expressed in retina via a tissue-specific promoter within intron 1 of the CtBP2 (C-terminal binding protein 2) gene,^40^ is a major structural protein of rod/cone synaptic contacts and delineates the OPL of controls (Fig. 4, row C). RIBEYE is not a transmembrane protein, and its transport to the synaptic terminal (the microtubule plus end) is not expected to be dependent on dynein. Mutant photoreceptor synaptic termini have normal levels of RIBEYE at P14 and P16 but are nearly devoid of CtBP2/RIBEYE at P21 (Fig. 4, row D), consistent with attenuated scotopic electroretinography (ERG) measurements. RIBEYE-immunoreactive puncta of the P21 OPL likely belong to cone pedicles, as the photopic ERG is near normal at P21. RIBEYE is undetectable at P30, consistent with absence of functional photoreceptors and “flat” rod and cone ERG amplitudes.

### Deletion of DYNC1H1 by Tamoxifen Induction

**Figure 5.**
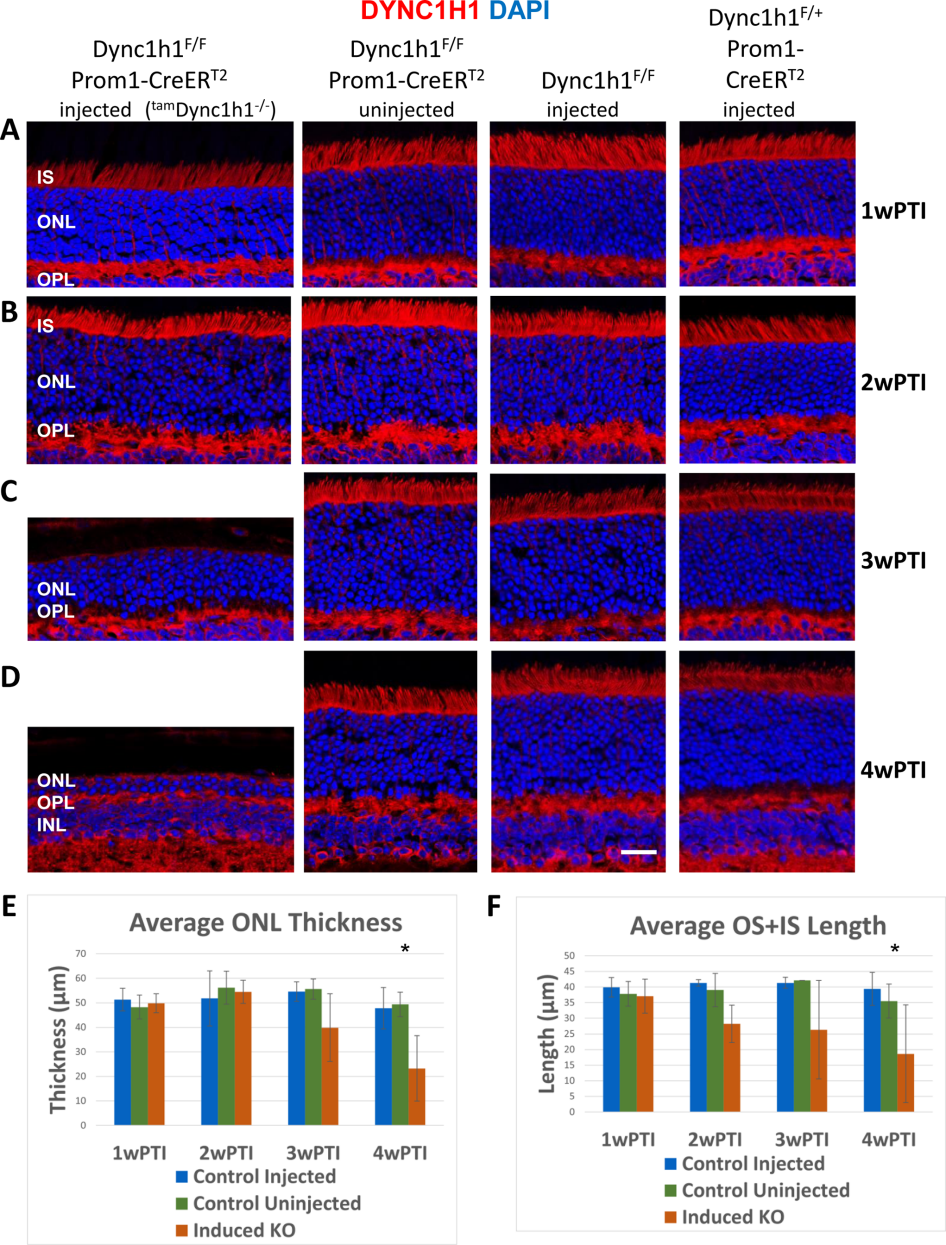
Tamoxifen-induced DYNC1H1 depletion in the adult retina. (**A–D**) ^tam^*Dync1h1*^−/−^(tamoxifen-induced KO, *first column*), uninjected *Dync1h1*^F/F^;Prom1-CreER^T2^ (*second column*), tamoxifen-injected *Dync1h1*^F/F^ retinas (*third column*), and tamoxifen-injected *Dync1h1*^F/+^;Prom1-CreER^T2^ (*fourth column*) harvested at 1wPTI (A), 2wPTI (B), 3wPTI (C), or 4wPTI (D). Cryosections were labeled with anti-DYNC1H1 (*red*) and contrasted with DAPI (*blue*). Uninjected *Dync1h1*^F/F^;Prom1-CreER^T2^ and injected *Dync1h1*^F/F^ or *Dync1h1*^F/+^;Prom1-CreER^T2^ retinas all maintain normal IS and ONL thickness throughout the experiment. In ^tam^*Dync1h1*^−/−^ (induced KO) retina, the ONL and IS shrank between 2wPTI and 3wPTI, but this shrinking was not statistically significant until 4wPTI. In retinas with efficient knockout of Dync1h1, ONL thinning increased in severity by 4wPTI. (**E**) Plot of average central ONL thickness at 1, 2, 3, and 4wPTI showing ONL thickness decreases in the ^tam^*Dync1h1*^−/−^ from 2wPTI to 4wPTI. *P < 0.05 (Welch's ANOVA). (**F**) Plot of average combined OS + IS lengths shows that ^tam^*Dync1h1*^−/−^ rod and cone OS shrink between 2wPTI and 4wPTI. *P < 0.05 (Welch's ANOVA). For E and F, the numbers of mice for control injected, control uninjected, and ^tam^*Dync1h1*^−/−^, respectively, were 1wPTI, *n* = 5, 5, 5; 2wPTI, *n* = 3, 3, 3; 3wPTI, *n* = 5, 5, 4; 4wPTI, *n* = 11, 5, 6.

**Figure 6.**
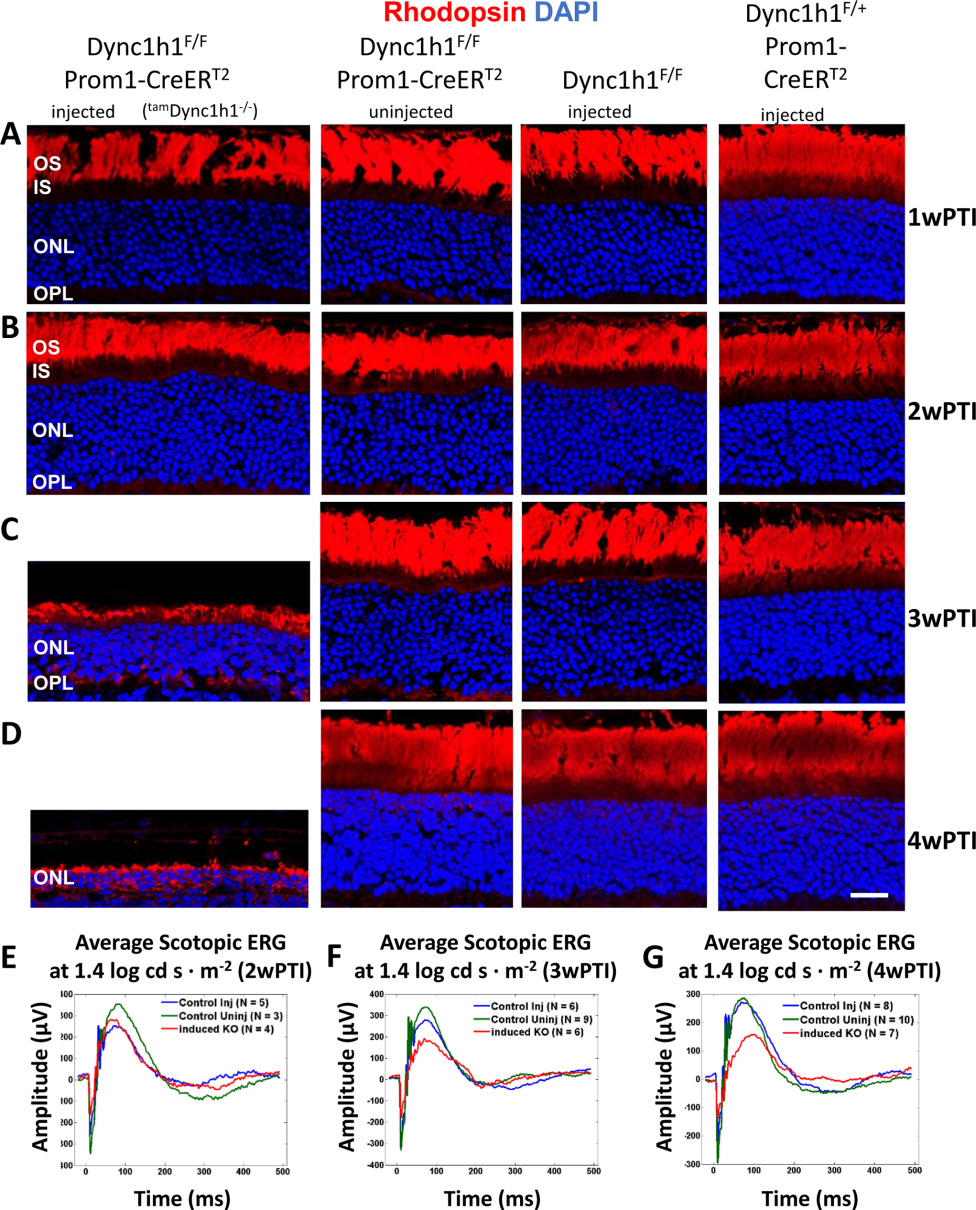
Effect of tamoxifen-induced DYNC1H1 deletion on rhodopsin expression. (**A–D**) Representative immunohistochemistry from ^tam^*Dync1h1*^−/−^ (*first column*), uninjected *Dync1h1*^F/F^;Prom1-CreER^T2^ (*second column*), tamoxifen-injected *Dync1h1*^F/F^ retinas (*third column*), and tamoxifen-injected *Dync1h1*^F/+^;Prom1-CreER^T2^ (*fourth column*) harvested at 1wPTI (A), 2wPTI (B), 3wPTI (C), or 4wPTI (D) and probed with anti-rhodopsin (*red*). Nuclei were contrasted with DAPI (*blue*). ^tam^*Dync1h1*^−/−^ retina reveals rod OS shortening and ONL thinning from 2 to 3wPTI. Rod OS shortening and ONL thinning increased in severity by 4wPTI, with minor rhodopsin expression in the ONL. (**E–G**) Average scotopic ERG traces at 1.4 log cd s·m^−2^ from control and ^tam^*Dync1h1*^−/−^ at 2wPTI (E), 3wPTI (F), and 4wPTI (G). Scotopic responses decrease between 2 and 4 wPTI in the ^tam^*Dync1h1*^−/−^.

To explore consequences of DYNC1H1 deletion in the adult mouse, we deleted *Dync1h1* in *Dync1h1*^F/F^;Prom1-CreER^T2^ 1-month-old mice using tamoxifen induction. Prom1 expression in adult mice is seen in rods and cones along with cells within the brain, pancreas, intestine/colon, kidney, lung, and reproductive system (male and female).^41^ *Dync1h1*^F/F^;Prom1-CreER^T2^, *Dync1h1*^F/F^, and *Dync1h1*^F/+^;Prom1-CreER^T2^ mice were injected with tamoxifen for 5 consecutive days, with the first injection occurring between P21 and P30 (Figs. 1C, D). Uninjected *Dync1h1*^F/F^;Prom1-CreER^T2^ mice served as an additional control. Injected *Dync1h1*^F/F^;Prom1-CreER^T2^ mice are abbreviated as ^tam^*Dync1h1*^−/−^ (Figs. 5, 6). Intraperitoneal injection of tamoxifen for 5 consecutive days induced the nuclear translocation of *Cre*, and the degeneration rate was assessed in retina cryosections of eyes harvested 1, 2, 3, and 4 weeks after the first injection. At 1 and 2 weeks post tamoxifen induction (wPTI), DYNC1H1 levels were indistinguishable among injected ^tam^*Dync1h1*^−/−^, uninjected *Dync1h1*^F/F^;Prom1-CreER^T2^, and injected *Dync1h1*^F/F^ or *Dync1h1*^F/+^;Prom1-CreER^T2^ mice (Fig. 5, rows A and B). At 3wPTI, DYNC1H1 was nearly eliminated in half of the ^tam^*Dync1h1*^−/−^ mice (see Fig. 5 legend). In ^tam^*Dync1h1*^−/−^ mice in which elimination of DYNC1H1 was seen, the ONL thickness was reduced by 50% and the OS + IS length was reduced to ∼30% that of control (Figs. 5C, E). In the other half of the ^tam^*Dync1h1*^−/−^ mice, tamoxifen-induced knockout of DYNC1H1 was inefficient and ONL and OS + IS measurements were normal or slightly reduced. DYNC1H1 antibody fluorescence in the OPL was undisturbed as DYNC1H1 levels in bipolar cells were unaffected in the tamoxifen-induced knockout (Fig. 5C). At 4wPTI, ONL thickness was reduced by 80% in mice with efficient tamoxifen injections, but DYNC1H1 was still detectable in the deteriorating mutant IS (Fig. 5D, E), reflecting failure to clear DYNC1H1 completely. In these mice, the average OS + IS length was reduced to about 12% of control (Fig. 5D, F).

**Figure 7.**
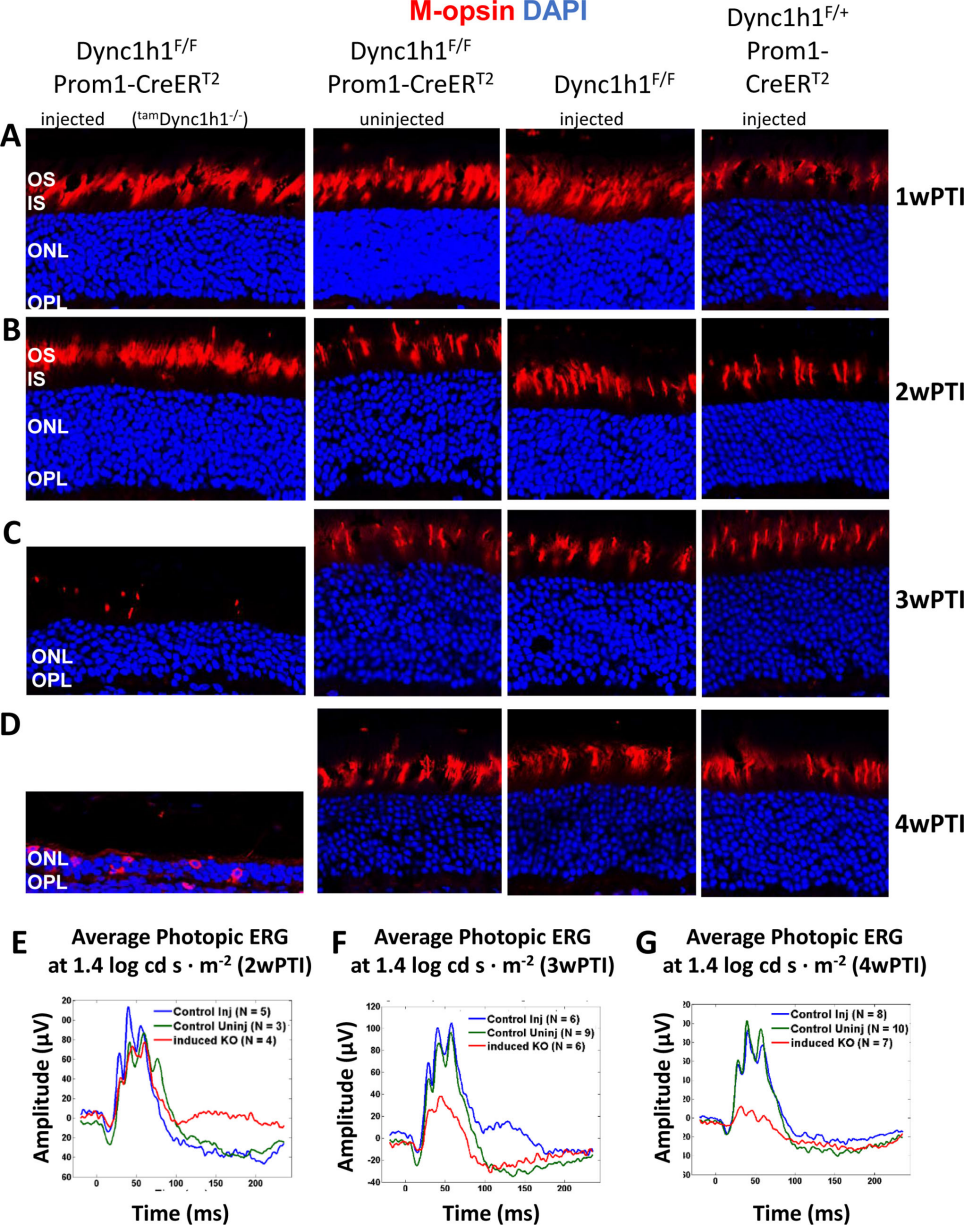
Effect of tamoxifen-induced DYNC1H1 deletion on cone-opsin expression. (**A–D**) Representative immunohistochemistry from ^tam^*Dync1h1*^−/−^ (*first column*), uninjected *Dync1h1*^F/F^;Prom1-CreER^T2^ (*second column*), tamoxifen-injected *Dync1h1*^F/F^ retinas (*third column*), and tamoxifen-injected *Dync1h1*^F/+^;Prom1-CreER^T2^ (*fourth column*) harvested at 1wPTI (A), 2wPTI (B), 3wPTI (C), or 4wPTI (D) probed with anti–M-opsin (*red*). ONL indicated by DAPI (*blue*). ^tam^*Dync1h1*^−/−^ retina reveals loss/shortening of cone OS from 2 to 3wPTI. While some M-opsin is seen in the ONL at 4wPTI, cone OSs are absent. (**E–G**) Average photopic ERG traces at 1.4 log cd s·m^−2^ from control and ^tam^*Dync1h1*^−/−^ at 2wPTI (E), 3wPTI (F), and 4wPTI (G). Photopic responses decrease between 2wPTI and 4wPTI in the ^tam^*Dync1h1*^−/−^.

As observed for DYNC1H1 at 1wPTI and 2wPTI, rhodopsin and M-opsin levels were indistinguishable among injected ^tam^*Dync1h1^−^^/^^−^*, uninjected *Dync1h1*^F/F^*;*Prom1*-*CreER^T2^, and injected *Dync1h1*^F/F^ or *Dync1h1*^F/+^;Prom1-CreER^T2^ mice (Figs. 6, 7, rows A and B). At 3wPTI in mice with efficient tamoxifen injections, ^tam^*Dync1h1*^−/−^ rod OS lengths were dramatically reduced and rhodopsin mislocalized in part to the IS and OPL (Fig. 6C, left column). Additionally, very few ^tam^*Dync1h1*^−/−^ cone OSs were retained (Fig. 7C). Almost no rhodopsin or M-opsin accumulated at the perinuclear endoplasmic reticulum (ER) of ^tam^*Dync1h1*^−/−^ photoreceptors at 3wPTI (Figs. 6C, 7C). At 4wPTI, the mutant ONL was reduced to two to three rows of nuclei containing traces of rhodopsin and M-opsin (Figs. 6D, 7D). Average scotopic electroretinography traces at 1.4 log cd s·m^−2^ (log candela seconds per square meter) at 2wPTI were nearly identical (Fig. 6E). At 3wPTI, scotopic a- and b-wave amplitudes were reduced in the ^tam^*Dync1h1^−^^/^^−^* retinas, compared to control injected or uninjected specimens (Fig. 6F). The reduction in scotopic a- and b-wave amplitudes of ^tam^*Dync1h1^−^^/^^−^* mice was even more evident at 4wPTI (Fig. 6G). Similarly, average photopic b-wave amplitudes of tamoxifen-induced knockout mice were comparable at 2wPTI (Fig. 7E) but reduced at 3wPTI (Fig. 7F). The photopic a- and b-wave amplitude of ^tam^*Dync1h1^−^^/^^−^* mice was nearly extinguished at 4wPTI (Fig. 7G).

**Figure 8.**
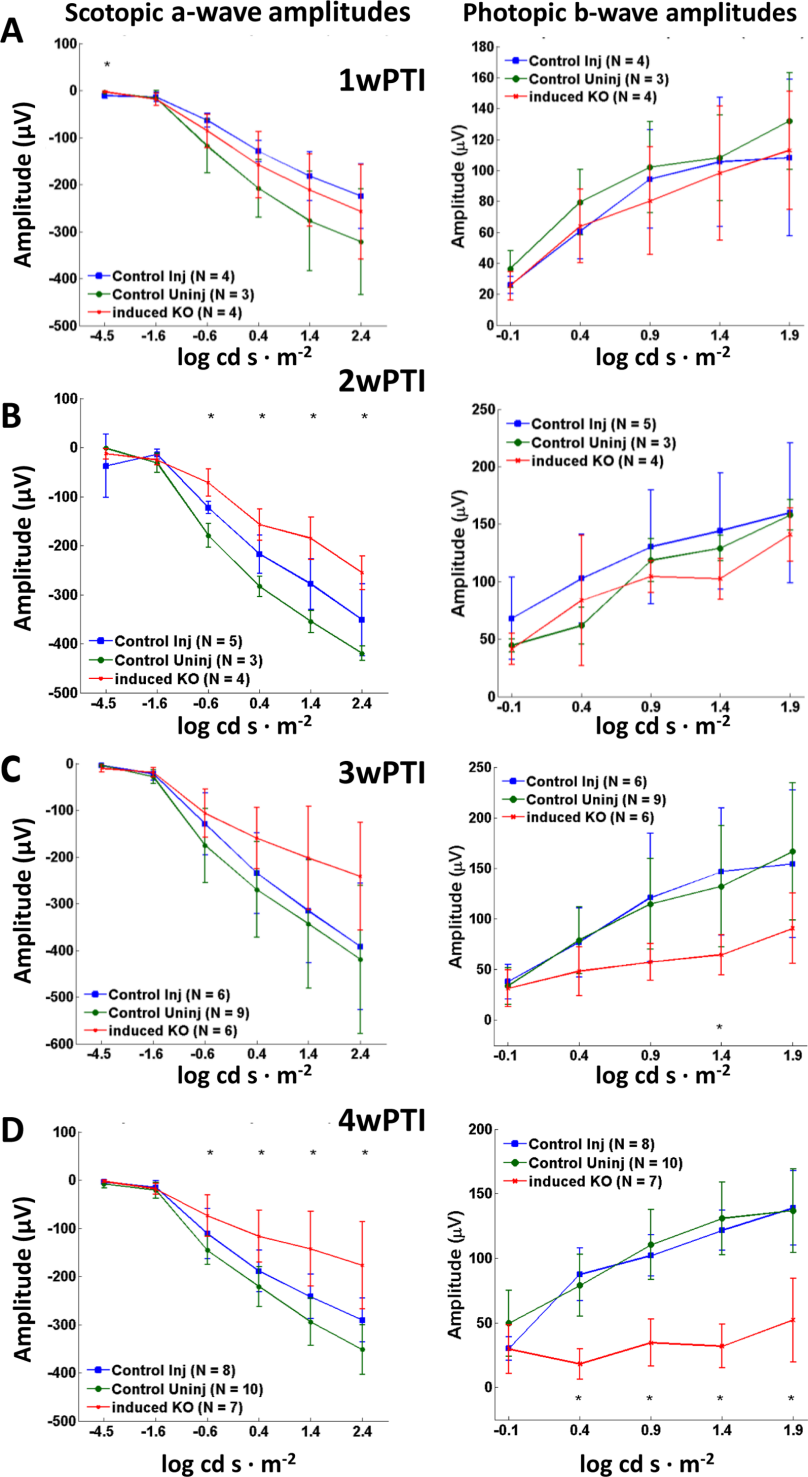
Summary of ERG responses as a function of intensity in ^tam^*Dync1h1*^−/−^ retina. (**A–D**) Average scotopic a-wave amplitudes (*left column*) and photopic b-wave amplitudes (*right column*) as a function of light intensity shrink between 2wPTI and 4wPTI. Injected control (*blue*), uninjected control (*green*), and tamoxifen-induced knockout (*red*). At 1wPTI (A), average scotopic and photopic ERGs amplitudes did not differ significantly between induced knockout and control. At 2wPTI (B), the average scotopic a-wave amplitude was slightly reduced compared to controls. Photopic ERGs did not differ. At 3wPTI (C), induced KO shows reduced average ERG amplitudes for both scotopic a-wave and photopic b-wave, but this reduction was only statically significant for the photopic 1.4 log cd s·m^−2^ intensity. At 4wPTI (D), the reduction in average ERG amplitude for both scotopic a-wave and photopic b-wave for the induced KO was statically significant for most ERG flash intensities. *P < 0.05 (unbalanced two-factor ANOVA, Tukey's honestly significant difference criterion).

Scotopic a-wave and photopic b-wave amplitudes as a function of light intensity did not display significant changes at 1wPTI for any of the light intensities (Fig. 8A). At 2wPTI, induced knockout (KO) did show as statistically different from control uninjected mice but not from the control injected mice. Control injected and control uninjected did not differ from each other at 2wPTI (Fig. 8B). At 3wPTI, scotopic a-wave and photopic b-wave amplitudes in the induced KO were reduced, but this reduction was only statistically significant at the photopic 1.4 log cd s·m^−2^ intensity (Fig. 8C). At 4wPTI, the reduction in scotopic a-wave and photopic b-wave amplitudes for induced KO was significant at most intensities (Fig. 8D). These results indicate that in mice in which loss of DYNC1H1 was efficiently induced by tamoxifen injection, rod and cone degeneration began between 2 and 3wPTI, with further retinal degeneration occurring between 3 and 4wPTI.

## Discussion

In our previous study,^13^ we conditionally knocked out DYNC1H1 in retina using Six3Cre that initiates ablation of DYNC1H1 after embryonic day 9. We showed that segregation of the ONL/INL was absent at P6 and P8, and mutant photoreceptors did not elaborate inner and outer segments. Here, we show that deletion of DYNC1H1 in mouse photoreceptors after ciliogenesis led to loss of function (Fig. 2), shortening of the inner and outer segments, and shrinking of the ONL (Figs. 3, 4). In wild-type (WT) photoreceptors, dynein, in conjunction with dynactin, transports cargo toward the minus end of microtubules at the MTOC or basal body (for a recent review, see Dahl and Baehr^14^). Once the basal body has docked to the IS cortex, inner segment biosynthetic activity is directed toward synthesis of outer segment proteins, transport of OS proteins to the periciliary ridge by dynein, movement through the connecting cilium either by diffusion or intraflagellar transport, and incorporation into nascent discs. As mouse photoreceptor outer segments are renewed every 10 days,^42^^,^^43^ sustained delivery of large amounts of transmembrane and peripheral membrane–attached and soluble proteins is essential.

When dynein is absent in photoreceptors before ciliogenesis (<P6), the ONL never forms correctly, and OS/synaptic structures are absent.^13^ In *Dync1h1* rod knockouts (this communication), the process was interrupted in the second postnatal week (>P16) (Figs. 3B, D; Figs. 4B, D). Interruption occurred in tamoxifen-induced knockouts between the second and third week postinduction (Figs. 5C, D; Figs. 6C, D). In the absence of dynein, delivery of OS proteins ceases and disc morphogenesis declined. In rod knockouts beginning at P16, ^rod^*Dync1h1*^−/−^ IS/OS shortened and were lost by P30, consistent with supply chain interruption of the secretory pathway. OS structures became unstable and essentially died by starvation. In tamoxifen-induced knockouts, rods and cones appeared to be stable for 2 weeks postinduction. As observed previously,^13^ impaired DYNC1H1 protein clearance delayed the onset of degeneration in rod (Fig. 3, row C) and tamoxifen-induced (Figs. 5C, D) knockouts of *Dync1h1*.

While Six3Cre-mediated excision of *Dync1h1* exons 24 and 25 had occurred by P6, DYNC1H1 clearance was slow and persisted past P16.^13^ In our rod knockout, excision of exons 24/25 was nearly complete at P10, but degeneration was not evident before P16, as judged by ONL thickness (Fig. 3E), presumably due to slow DYNC1H1 clearance. Similarly, in our tamoxifen induction experiments, DYNC1H1 persisted for 2 weeks after the initial intraperitoneal injection before onset of degeneration. In contrast to retina- and rod-specific deletion of DYNC1H1, the efficiency of tamoxifen induction was variable. For example, at 4wPTI, OS + IS length was severely reduced to 12% of control when tamoxifen induction was efficient but remained 88% that of control when induction was inefficient (Fig. 5F). However, even in mice in which tamoxifen induction was inefficient, ONL thickness was reduced by 30%, likely reflecting a partial loss of DYNC1H1. These variances in the efficiency of tamoxifen induction were likely due to tamoxifen not being completely in solution.

The most abundant OS membrane protein, rhodopsin, is synthesized at the endoplasmic reticulum, posttranslationally modified in the Golgi, and transported to the OS base via the secretory pathway.^11^^,^^44^ Newly synthesized rhodopsin is predicted to accumulate in the ONL in the absence of dynein, as COPII vesicle transport from the ER to Golgi is impaired. Unexpectedly, we observed little rhodopsin accumulation or mislocalization in the ^rod^*Dync1h1*^−/−^ ONL (Fig. 3D), an observation that could mean that rhodopsin biosynthesis terminates. Alternatively, newly synthesized rhodopsin may be cleared immediately if it cannot exit the ER or if rhodopsin-containing vesicles cannot attach to dynein after ER exit.

PDE6 is a key protein in the phototransduction cascade regulating the levels of cGMP, the internal transmitter of phototransduction.^45^ Rod PDE6 is peripherally membrane attached using prenyl side chains as anchors.^39^ PDE6D (PDEδ) acts as a trafficking chaperone to move PDE6 to the OS by diffusion.^46^ In the absence of PDE6D, PDE6 still manages to reach the OS, presumably hitching a ride with rhodopsin-bearing vesicles in the secretory pathway powered by dynein.^47^ It is unclear whether PDE6 transport in the absence of PDE6D depends on dynein. Mouse RIBEYE is a ribbon-specific protein, expressed under its own promoter within the *CtBP2* gene.^40^ It has the capability to build the synaptic ribbon scaffold via multiple RIBEYE–RIBEYE interactions. The B domain of RIBEYE is a homolog of 2-hydroxyacid dehydrogenase, binding NAD^+^^40^ and UNC119.^48^ RIBEYE is presumably not a membrane-associated protein, and while its transport mode to the synaptic region is unknown, it is not likely to be dynein dependent. Both PDE6 and RIBEYE disappear as rod photoreceptors degenerate rapidly.

Our study currently is limited to evaluation of photoreceptor degeneration by confocal microscopy and electroretinography. We show that photoreceptor OS, IS, and ONL nearly simultaneously shrink in rod knockouts beginning at P16 and in tamoxifen-induced knockouts by the third week postinjection. OS likely shrink by failed delivery of newly synthesized proteins to the periciliary ridge near the basal body. It will be interesting to evaluate the status of the IS microtubule cytoskeleton in the absence of dynein by immunohistochemistry with anti-tubulin antibodies, the effect on disc morphogenesis as protein delivery ceases, and potential changes in synaptic structures by transmission electron microscopy.

## Methods

### Animals

All procedures were approved by the University of Utah Institutional Animal Care and Use Committee (Protocol 18-11005). Prom1^tm1(cre/ERT2)Gilb^ (Stock No: 017743) and Egfp-*Cetn2* mice Stock No. 008234–CB6-Tg(CAG-EGFP/CETN2)3-4Jgg/J) were obtained from The Jackson Laboratory. iCre75 mice were generated in Utah.^34^ *Dync1h1*^F/F^ mice have been described.^13^

### Generation of Rod Knockout Mice

*Dync1h1* ^F/F^ mice^13^ were crossed with iCre75 transgenic mice^34^ kept on an Egfp-Cetn2 background to generate *Dync1h1* ^F/+^;iCre75;Egfp-*Cetn2* mice. Mice were then backcrossed to *Dync1h1*^F/F^ to generate experimental animals. Expression of Egfp-Cetn2 allows connecting cilium and centriole identification without use of a specific antibody.

### Generation of Knockouts by Tamoxifen Induction

*Dync1h1* ^F/F^ mice were bred to Prom1-CreER^T2^ mice^41^ to generate *Dync1h1* ^F/+^;Prom1-CreER^T2^ mice. In these mice, expression of CreER^T2^ is driven by the prominin 1 (Prom1) promoter/enhancer. *Dync1h1* ^F/+^;Prom1-CreER^T2^ mice were bred to *Dync1h1*^F/F^ mice to generate mice for tamoxifen experiments. Tamoxifen was administered via intraperitoneal injection in adult mice (P21–P30 at time of first injection). Tamoxifen was dissolved in corn oil to a stock solution of 20 mg/mL. Mice were dosed with 150 mg/kg body weight for 5 consecutive days according to their weight on the first day of injections (i.e., 7.5 µL of 20 mg/mL tamoxifen solution per gram weight). ERG and collection of eyes for confocal immunolocalization were performed 1 to 4 weeks after the first injection.

### Genotyping

Genomic DNA was extracted from fresh tissue by dissolving tail clips from P4 to P14 mice in 200 µL tail lysis buffer at 50°C to 60°C for 1 to 2 hours. Digests were then centrifuged at 15,000 rpm for 5 minutes. Supernatant was added to an equal volume of isopropanol and centrifuged at 15,000 rpm for 5 minutes. Pellet was rehydrated in 100 µL H_2_O. Genotyping was performed by PCR with EconoTaq DNA polymerase (Lucigen, Middleton, WI). To verify the deletion of exons 24 and 25 of *Dync1h1* in ^rod^*Dync1h1*^−/−^ retina, P10 to P21 retinal genomic DNA was extracted and amplification was performed with primers exon 23-F (5′-TCTCTGGAAAGGTTGGCAGA) and loxP-R (5′-GAGATCAGTTGCGGTTTGCTAGT). Amplicon sizes were *Dync1h1* WT allele (1.3 kb), *Dync1h1*^F^ allele (1.6 kb), and Cre-recombination allele (500 bp) (Fig. 1E). Primers flanking the loxP site of introns 25 to 26 were used to distinguish between wild-type and floxed *Dync1h1* alleles (loxP-F, 5′-TGATGGTCTTGGCTAATTGGTGG; loxP-R, 5′-GAGATCAGTTGCGGTTTGCTAGT) with amplicon sizes of 269 bp (floxed) and 202 bp (WT) (Fig. 1F). Presence of iCre75 transgene was verified with iCre75-F (5′-GGATGCCACCTCTGATGAAG) and iCre75-R (5′-CACACCATTCTTTCTGACCCG) primers (amplicon size 700 bp) (Fig. 1G). Presence of EGFP-CETN2 was determined by PCR using primers Cetn2-F (5′-TGAACGAAATCTTCCCAGTTTCA) and Cetn2-R (5′-ACTTCAAGATCCGCCACAACAT) (amplicon size 600 bp) (Fig. 1H). Presence of Prom1-CreER^T2^ was verified with Prom1-F (5′-CAGGCTGTTAGCTTGGGTTC) and Prom1-CreER^T2^-R (5′-AGGCAAATTTTGGTGTACGG) primers (amplicon 320 bp) with Prom1-F and Prom1-WT-R (5′-TGCTGATTGCCTTCTGTCTG) (amplicon 586 bp) serving as a control (Fig. 1I).

### Eye Collection Methods

Mice were sacrificed by cervical dislocation. Eyes were enucleated and immersion-fixed for 1 hour using 4% paraformaldehyde in 0.1 M phosphate buffer, pH 7.4. Anterior segments were removed after 10 minutes of fixation. Eyecups were equilibrated in 15% sucrose in PBS, equilibrated overnight in 30% sucrose in PBS, embedded in Optimal Cutting Temperature (OCT) compound, frozen on dry ice, and stored at −80°C. Blocks containing eyecups were equilibrated to −20°C prior to sectioning. Transverse sections, including or adjacent to the optic nerve, were cut at a 14-µm thickness using a (Leica, Buffalo Grove, IL) cryostat and transferred to charged slides (Thermo-Fisher, Waltham, Massachusetts). Slides were stored at −80°C. Control retinas included *Dync1h1* ^F/F^, *Dync1h1* ^F/+^, or *Dync1h1* ^F/+^;iCre75 littermates for ^rod^*Dync1h1*^−/−^ experiments. Control retinas for ^tam^*Dync1h1*^−/−^ experiments were uninjected *Dync1h1* ^F/F^;Prom1-ETCre, injected *Dync1h1* ^F/F^, and injected *Dync1h1*^F/+^;Prom1-CreER^T2^ mice.

### Immunohistochemistry

Sections were encircled using a PAP pen (Ted Pella, Redding, CA), warmed for 30 minutes at 37°C, and then rehydrated by washing 10 minutes three times in 1× PBS. All sections used were from the central retina near the optic nerve. Sections were blocked in 5% normal goat serum/0.1% TritonX-100 in 1× PBS for 1 hour. Antibodies, dilutions, and sources follow: DYNC1H1 (dilution 1:250, 12345-1-AP; Proteintech, Proteintech Rosemont, IL),^4^^,^^49^ rhodopsin (1:1000, A15093; Abclonal, Abclonal Woburn, MA), PDE6 (1:1000; MOE Cytosignal, Cytosignal IRVINE, CA),^50^ OPN1MW/OPN1LW (1:500, AB5405; Millipore Sigma, Millipore Sigma St. Louis, MO),^50^^,^^51^ and CtBP2/RIBEYE (1:10,000, 612044; BD Biosciences, BD Biosciences Franklin Lakes, NJ). Primary antibodies were diluted in blocking buffer and applied to sections; sections were then incubated overnight at 4°C. Slides were washed for 10 minutes three times in PBS. Secondary antibodies were diluted in blocking buffer (goat anti-rabbit Alexa Fluor 555, 1:1000 [32732; Invitrogen, Invitrogen Waltham Massachsetts]; goat anti-mouse Alexa Fluor 555, 1:1200 [32737; Invitrogen]; Invitrogen Waltham Massachsetts DAPI, 1:5000), applied to the sections, and sections were incubated in the dark for 1 hour at room temperature. Slides were washed for 10 minutes three times in PBS. Slides were dipped briefly in deionized H_2_O and coverslipped using Fluoromount-G Mounting Medium (Southern Biotech, Southern Biotech Birmingham, AL). Images were acquired using the 40× objective of a Zeiss (Zeiss Jena 07745, Germany) LSM800 confocal microscope. All genotypes of a given age and antibody were imaged at a single z-plane using identical settings for laser intensity and master gain. Digital gain was 1 for all images. Pinhole size was set for 1 AU on the red channel (39 µm for the 40× objective). Postprocessing of nonsaturated images consisted of equal adjustments to brightness and contrast of control and knockout images using Adobe Photoshop (Adobe Photoshop San Jose, CA) but without affecting the conclusions made.

### Electroretinography

Scotopic and photopic ERG measurements were performed using P16, P21, or P30 mice for the iCre75 experiments and at 1, 2, 3, or 4 weeks after tamoxifen induction for the Prom1-CreER^T2^ experiments. Prior to ERG, the mice were dark-adapted overnight and anesthetized with an intraperitoneal injection of 1% ketamine/0.1% xylazine at 10 µL/g body weight. The mice were kept warm during ERG by using a temperature-controlled stage. Scotopic and photopic responses were recorded as described^52^ using a UTAS BigShot Ganzfeld system (LKC Technologies, Gaithersburg, MD, USA). Scotopic single-flash responses were recorded at stimulus intensities of −4.5 to 2.4 log cd s·m^−2^. Mice were light-adapted under a background light of 1.48 log cd s·m^−2^ for 5 to 10 minutes prior to measuring photopic responses. Photopic single-flash responses of control and knockout were recorded at stimulus intensities of −0.1 to 1.9 log cd s·m^−2^.

### Statistical Analysis

We performed an unbalanced two-factor ANOVA to compare experimental and control animals for their quantified a- and b-wave ERG response across multiple ages or weeks after tamoxifen induction. Post hoc multiple comparison was performed using Tukey's honestly significant difference criterion. Statistical significance was determined using an α value of *P* < 0.05. All electroretinography statistics were computed using statistical toolbox “anovan” and “multcompare” functions in MATLAB (MathWorks, Natick, MA, USA).

We performed a Student's *t*-test assuming unequal variances to compare iCre75 control and KO ONL thickness or IS + OS length. Retina measurements used for these calculations were determined based on an average of three measurements per retina. Microsoft Excel (Microsoft, Redmond, WA, USA) function “T.TEST assuming unequal variances” was used to calculate the *P* value, with statistical significance determined using an α value of *P* < 0.05. Since we had unequal sample sizes and could not assume homogeneity of variances, we preformed Welch's ANOVA test to compare control injected, control uninjected, and ^tam^*Dync1h1*^−/−^ ONL thickness or IS + OS length using the Xrealstats data analysis add-on to Microsoft Excel. Statistical significance was determined using a *P* value of <0.05.
